# Distribution and habitat database of fluvial Plecoptera, Trichoptera and Coleoptera from Sierra Nevada, Spain

**DOI:** 10.1038/s41597-024-03652-y

**Published:** 2024-07-24

**Authors:** Manuel Jesús López-Rodríguez, Andrea Ros-Candeira, María del Carmen Fajardo Merlo, Marta Sáinz Bariáin, Carmen Elisa Sainz-Cantero Caparrós, José Manuel Tierno de Figueroa, Carmen Zamora-Muñoz

**Affiliations:** 1 Department of Ecology, Faculty of Sciences, University of Granada, Avenida Fuente Nueva, s/n, 18071 Granada, Spain; 2 Laboratory of Ecology, Andalusian Institute for Earth System Research (IISTA-CEAMA), University of Granada, Avenida del Mediterráneo s/n, 18006 Granada, Spain; 3 Environment and Water Agency. Department of Sustainability, Environment and Blue Economy. Regional Government of Andalusia, C/ Minerva 7. Edificio Zeus III, local, 18014 Granada, Spain; 4 Spanish Institute of Oceanography, Spanish National Research Council (IEO-CSIC), Santander Oceanographic Center (COST-IEO). Avenida de Severiano Ballesteros, 16, 39004 Santander, Spain; 5 Department of Zoology, Faculty of Sciences, University of Granada, Avenida Fuente Nueva, s/n, 18071 Granada, Spain

**Keywords:** Biodiversity, Entomology

## Abstract

Sierra Nevada (southern Iberian Peninsula) harbours a great biodiversity and the studies on some aquatic insect groups have been and continue to be numerous there. This database brings together information on Plecoptera, Trichoptera and Coleoptera inhabiting running waters of this mountain system above 800 m of altitude. It includes data on the number, life stage and sex of individuals as well as the available information on abiotic characteristics of their habitats. The dataset is composed of 1,718 sampling events carried out between 1901 and 2022 in approximately 60 different water bodies, 15,347 occurrences pertaining to more than 203,000 individuals, and 10,173 records of associated measurements (23 physico-chemical parameters). The dataset is the result of a comprehensive review of scientific literature and of integrating data from recent research projects and the Sierra Nevada Global-Change Observatory’s long-term monitoring data. This information is valuable for those studying past distributions and abundances of the species in the dataset, for building predictive models or just studying temporal trends in the current context of climate change.

## Background & Summary

The mountain system of Sierra Nevada has plenty of water courses, and its East-West alignment separates two clear faces, one at the North in which streams flow to the Guadalquivir Basin, and one at the South in which streams drain into several small basins reaching the Mediterranean Sea. Its altitudinal gradient (ranging from around 700–800 to 3479 m a.s.l.) supposes a great shift in conditions in a relatively small distance, so conditioning the assemblage of organisms that compose the communities of these streams and rivers^1^. This has promoted evolutionary and biogeographical processes that have led to the biocoenosis we observe today.

The National and Natural Park of Sierra Nevada is a recognised biodiversity hotspot both in the Mediterranean Basin and at global scale^2^. Its particular characteristics (aforementioned altitudinal gradient, the presence of five out of six of the Mediterranean bioclimatic zones, its location in the southernmost part of Europe, etc.) makes it a specially interesting mountain range to study and monitor, from which relevant information regarding climate change effects on particular organisms and ecosystems may arise. Specifically, freshwater ecosystems are particularly interesting from a conservation point of view. Worldwide, around 65% of the aquatic habitat supported by global river discharge is under moderate to high threat, and nearly 4.8 billion of the world’s population (for 2000) lives in areas where either incident human water security or biodiversity threat exceeds the 75th percentile^3^. Thus, human activities during the last two centuries have also influenced the conditions within these environments and, so, the composition of these communities. Particularly interesting to study these processes are organisms that are very sensitive to condition changes, such as some groups of aquatic macroinvertebrates and, specifically, aquatic insects. Thus, with the aim of long-term monitoring these fragile, high mountain ecosystems, the Sierra Nevada Global-Change Observatory started to develop a program to seasonally analyse the community of macroinvertebrates from several streams from Sierra Nevada. Besides that, several other studies have been accomplished in Sierra Nevada streams and rivers at different levels (community, population, etc.), with different purposes (biomonitoring, study of phenology, ecology, etc.), and in different animal groups. Many of them have been carried out in four main orders of insects, namely Ephemeroptera (mayflies), Plecoptera (stoneflies), Trichoptera (caddisflies) and Coleoptera (beetles), but also particular taxa of Diptera (midges and relatives) and the brown trout (*Salmo trutta*) have been studied (for a review, see^4^ and^1^).

Here, we present the *Dataset of Plecoptera, Trichoptera and Coleoptera from Sierra Nevada*^5^. In it, we have focused on fluvial species living above 800 m a.s.l. of three widely studied groups of insects that usually act as bioindicators, namely stoneflies (Plecoptera), caddisflies (Trichoptera) and aquatic beetles (Coleoptera). We have compiled information from both published and grey literature, from recent research projects and from the monitoring program carried out since 2007 by the Sierra Nevada Global-Change Observatory^6^.

## Methods

### Data acquisition

**Fig. 1:**
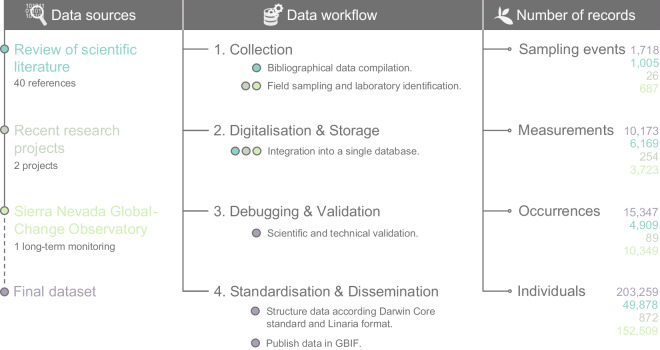
Dataset summary. From left to right: sources from which data were obtained, data workflow followed to compile the dataset and number of records (total and per source) of the Darwin Core.

Information used for the database comes from three different sources (Fig. 1): a bibliographic compilation made by the experts of each insect group (Table 1), data coming from ongoing or recently finished research projects (not yet published) and data from the long-term monitoring project carried out by the Sierra Nevada Global-Change Observatory. As all the existing information regarding the scope of the compilation, both published and grey literature, was already present in the personal library of each insect group expert participating in this work, no additional bibliographic searches were needed.

**Table 1:** Temporal range of the references used as data sources in the bibliographic compilation.

Publication year	References
1902	^20^
1930	^21^
1952	^22^
1954	^23^
1956	^24^
1962	^25^
1963	^26^
1967	^27^
1978	^28^
1979	^29^
1986	^30^
1988	^31^
	^32^
1989	^33^
	^34^
	^35^
1990	^36^
	^37^
1991	^38^
	^39^
1992	^40^
	^41^
	^42^
	^43^
1994	^44^
1998	^45^
2000	^46^
2002	^47^
	^48^
	^49^
2004	^50^
2008	^51^
	^52^
2010	^53^
	^54^
2013	^55^
2014	^56^
2018	^57^
2019	^58^
2021	^59^

The criteria for including information in the database were as follows, and applied for the three sources data, i.e., the bibliographic compilation, the data coming from ongoing or recently finished research projects and the Sierra Nevada Global-Change Observatory (Fig. 1). For the three insect orders, only records for which specific location data from running water ecosystems existed were included. In the case of Coleoptera, an additional selection criterion was applied: species included corresponded to the taxa that were specifically included in^7^ and were considered by^8^ as “mainly aquatic” (those in which at least 50% of the species that comprise them are linked to aquatic environments) and belong to the ecological group of “true aquatic beetles”, defined by^9^ as those in which the adult form remains totally or partially submerged most of the time. Although the members of the family Scirtidae do not fit the above criteria, they were also included as they were frequent elements in the records made by the Sierra Nevada Global-Change Observatory.

### Study area

**Fig. 2:**
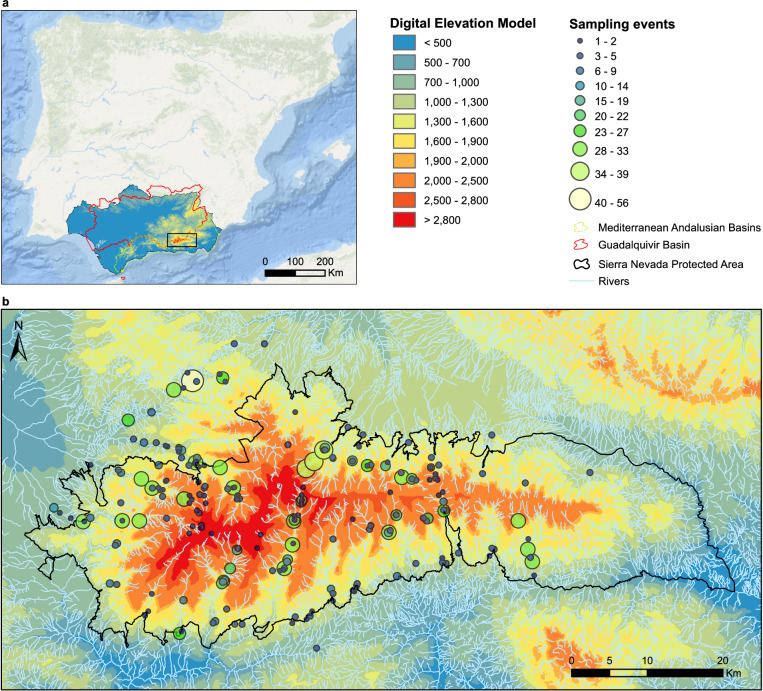
Map of the sampling event locations. (**a**) Bounding box of the study area. (**b**) Number of sampling events for each location. More than 98% of the sampling events in the dataset have coordinates, resulting in a total of 234 different locations.

**Fig. 3:**
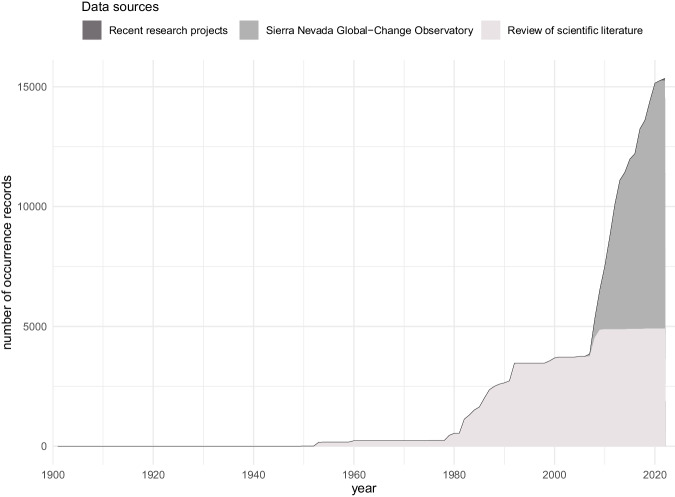
Cumulative number of occurrence records over time in the dataset. The curve grows suddenly from 2008 onwards, when the long-term monitoring of the Sierra Nevada Global-Change Observatory takes hold.

**Fig. 4:**
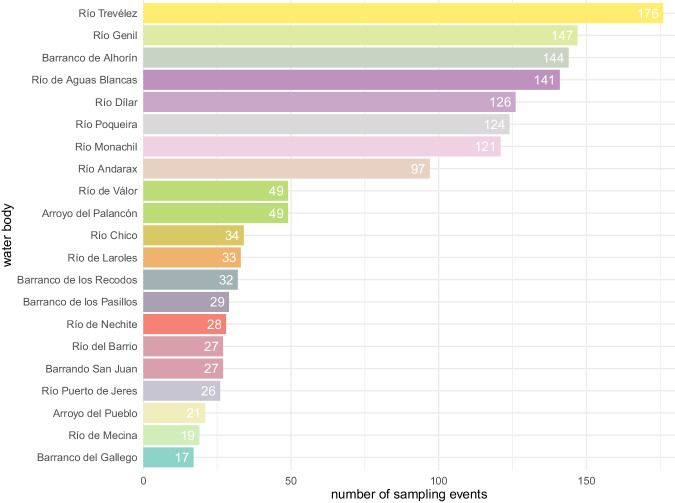
Number of sampling events per river. More than 60 water bodies were sampled, the figure only shows rivers with more than 15 sampling events.

The bibliographic information compiled for this database covered streams and rivers above 800 m of altitude within the Sierra Nevada massif (Granada and Almería, Spain) (Fig. 2). No lentic system was included. Records from 1901 to 2022 were included, but most of them came from the 1980’s in advance (Fig. 3). The collection of data from scientific papers covered studies published between 1902 and 2021 (in the ‘associatedReferences’ element of the database^5^ users can look up exactly which scientific paper each piece of data was collected from). From the first records to around 1980, most occurrences correspond to particular sampling campaigns of certain naturalists/researchers searching for organisms of particular groups (either Plecoptera, Trichoptera or Coleoptera). From around 1980 to 2008 the increase in occurrences coincide with the take-off and development of a research group in the University of Granada, to which several of the authors belong, dedicated to the study of aquatic insects, mostly in Sierra Nevada. Finally, from 2008 the increase is due to the program for long-term monitoring of the Sierra Nevada Global-Change Observatory. This information was enriched with data collected in a recently finished research project in three different sampling stations in the Poqueria stream (at approximately 1500 m, 2000 m and 2500 m a.s.l.) in 2022, data collected in one sampling station in the Válor stream (at approximately 1900 m a.s.l.) in 2022, and from samplings carried out within the long-term monitoring program of the Sierra Nevada Global-Change Observatory made in 23 sampling stations distributed in eight streams and rivers of Sierra Nevada (Alhorí, Andarax, Bayárcal, Dílar, Genil, Monachil, Poqueira, and Trevélez, the latter used also for studying the phenology of stoneflies) within the period 2007–2021. Figure 4 shows which are the most sampled rivers.

### Sampling protocol

Many different methodologies have been used in the studies from which data of the present database come from, depending on the kind of information that wanted to be acquired (quantitative or qualitative) and on the developmental stage that wanted to be studied (nymphs/larvae or adults). Broadly speaking, for the aquatic stages (nymphs for Plecoptera, larvae and pupae for Trichoptera and both adults and larvae for Coleoptera, depending on the species) a kick or hand net was used if authors wanted to collect qualitative data, and a Surber sampler (with different areas of sampling) was used if quantitative data wanted to be acquired.

In the case of terrestrial adults (in both Plecoptera and Trichoptera), a sweep net was used and/or direct collection from the stones, riparian vegetation, etc. Flying adults for some species of Trichoptera were captured with black light and actinic traps during emergence periods. These samplings were either qualitative, without defining an area or time of sampling, or quantitative, usually per unit of effort (sampling both shores in a given unit of time, e.g., 30 minutes, or over a certain distance, e.g., 50–100 m long on each shore).

Individuals were preserved in either 70% or 96% ethanol and identified in the laboratory to the maximum possible taxonomic resolution from morphological characters.

In the particular case of the information coming from a recently finished research project in the Poqueira and Válor streams, samplings of the whole community of macroinvertebrates were carried out using a kick net (0.25 m × 0.5 m), stirring the substrate situated at 0.5 m from the mouth of the net with the hand and the foot. Six samples were collected in each sampling station. Samples were preserved in 70% ethanol and brought to the laboratory for their sorting. Only those taxa that were going to be used for particular purposes (further nymphal biology studies) were separated in these streams and identified at the species level. In both streams, adults of stoneflies were collected from the stones and the riparian vegetation using tweezers and a sweep net, respectively, along the two shores of the sampled stream reach (approximately 50 m long each). These individuals were preserved in 70% ethanol and subsequently identified in the laboratory to the species level from morphological characters.

Data from the long-term monitoring program of streams and rivers carried out by the Sierra Nevada Global-Change Observatory were obtained using the standard protocol for monitoring wadeable streams in Spain^10^. Following this methodology, a 100 m stream reach was selected where the most frequent habitats were represented (hard substrates, plant detritus, banks with vegetation, submerged macrophytes, fine sediments). The covering (%) of the habitat types determined the number of samples (of a maximum of 20) that correspond to each habitat following a stratified design. At each sampling point, macroinvertebrate samples were taken with a kick net (0.25 m × 0.5 m), using the hands and feet to stir the substrate situated at 0.5 m from the mouth of the net. Once the samples were collected, the organisms were preserved in 96% ethanol. These samples were sorted out in the laboratory, separating Plecoptera, Trichoptera and Coleoptera from the rest, which were identified to the genus or species level, if possible, from morphological characters. In the case of monitoring the phenology of Plecoptera, qualitative and quantitative samples were taken, both of nymphs and adults, in the Trevélez river at three different altitudes (1500, 2000, and 2500 m a.s.l.). For the nymphs, a Surber net (40 × 40 cm) was used in six placements within the studied reach, while for adults, a transect (50 m long by 5 m wide) was sampled on both banks, where the riparian vegetation was beaten with an entomological rod while looking for stonefly adults hidden among the rocks. These adults were placed in 10-ml vials containing 70% ethanol to be separated and identified to the species level in the laboratory from morphological characters.

### Data management and standardization

After organising the information from all sources in a relational database, several custom-made SQL queries were applied to structure data according to the Darwin Core standard^11^. The resulting sampling-event dataset was published through the Integrated Publishing Toolkit^12^ (IPT v2.7.5) of the Spanish node of the Global Biodiversity Information Facility (GBIF) (http://ipt.gbif.es).

This collaborative framework to put together data from research and management, and to make the data available to the scientific community, managers, and the general public, responds to the Sierra Nevada Global-Change Observatory efforts to manage data following the FAIR principles^13^. In order to ensure the Findability (1), Accessibility (2), Interoperability (3), and Reusability (4) of the dataset we:

1. Integrate and disseminate data and metadata through GBIF with a unique and persistent identifier (DOI) assigned. Also integrate the data in the institutional data repository of the Sierra Nevada Global-Change Observatory^14^, which increases its findability by managers and local users.
2. Provide open and free access to the data and metadata, in line with the support and commitment of the European Commission concerning Open Science.
3. Standardise data to the Darwin Core and metadata to the Ecological Metadata Language (EML).
4. Apply a non-restrictive license and provide a well-documented description of the dataset in this data descriptor and GBIF metadata sections.

## Data Records

**Fig. 5:**
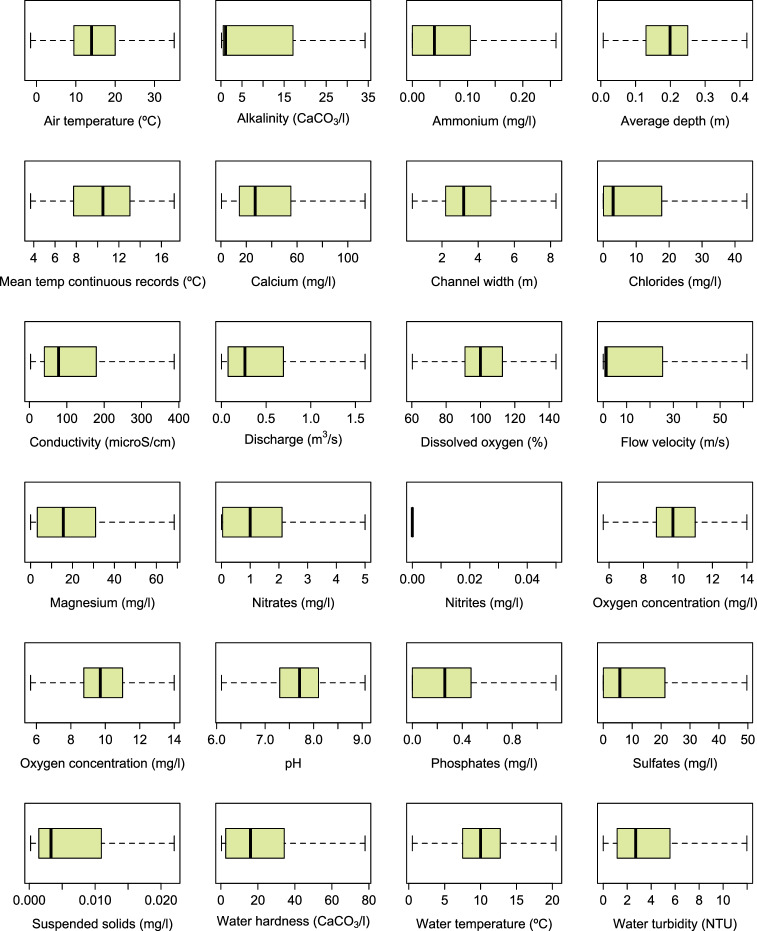
Box-plots representing the range of values of each physico-chemical parameter compiled in the database.

**Fig. 6:**
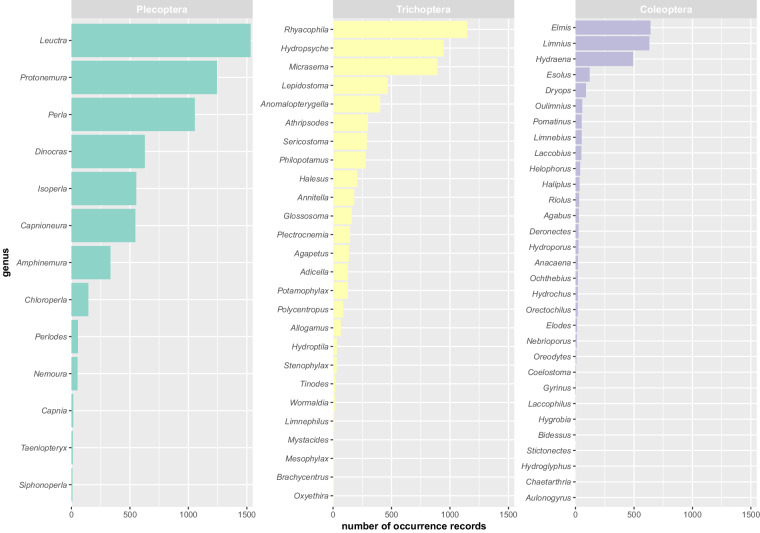
Number of occurrence records per genus, excluding records identified at only the family level.

**Fig. 7:**
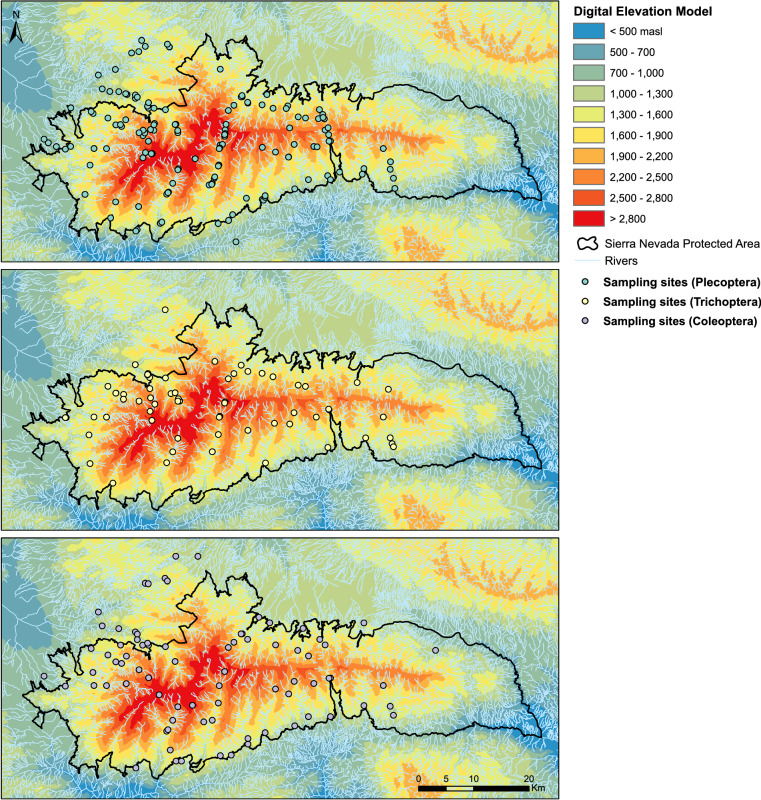
Map showing the sampling sites where individuals of each insect order were collected.

The data descriptor we present corresponds to version 1.6 of the dataset called *Dataset of Plecoptera, Trichoptera and Coleoptera from Sierra Nevada*^5^, which can be downloaded as a Darwin Core Archive (DwC-A) through GBIF (the repository website can be changed to English in the top right corner of the website). The dataset was standardised to the Darwin Core structure as sampling-event data. The DwC-A of this sampling-event dataset is composed of 3 files: the event core contains 1,718 sampling events carried out between 1901 and 2022 in approximately 60 different water bodies, the occurrence extension has 15,347 occurrences belonging to more than 203,000 individuals, and the measurement or facts extension contains 10,173 event-associated measurements of 23 physico-chemical parameters (water temperature, discharge, ammonium, phosphates, nitrites, pH, conductivity, dissolved oxygen, oxygen concentration, channel width, chlorides, nitrates, air temperature, alkalinity, calcium, magnesium, flow, velocity, water hardness, sulphates, average depth, suspended solids, water turbidity, and average water temperature from a continuous record; Fig. 5). The occurrence records belong to the Plecoptera, Trichoptera and Coleoptera orders. There are 30 families, 70 genera, 121 species and 12 subspecies represented in this dataset. Figure 6 shows the number of occurrence records for each genus of Plecoptera, Trichoptera and Coleoptera in the dataset (excluding data at the family level), and Fig. 7 the sampling sites where organisms were collected for each order.

The Darwin Core elements included in the Event Core are: eventID, modified, language, institutionCode, ownerInstitutionCode, datasetName, license, eventDate, year, month, day, continent, country, countryCode, waterBody, locality, locationRemarks, minimumElevationInMeters, maximumElevationInMeters, verbatimCoordinates, verbatimLatitude, verbatimLongitude, verbatimCoordinateSystem, decimalLatitude, decimalLongitude, coordinateUncertaintyInmeters, geodeticDatum, samplingProtocol, sampleSizeValue, sampleSizeUnit, samplingEffort, eventRemarks. For the Occurrence Extension are: occurrenceID, catalogNumber, collectionCode, eventID, eventDate, recordedBy, recordedByID, associatedReferences, organismRemarks, taxonRemarks, organismQuantity, organismQuantityType, lifeStage, sex, basisOfRecord, scientificName, taxonRank, kingdom, phylum, class, order, family, subfamily, genus, subgenus, specificEpithet, infraSpecificEpithet, scientificNameAuthorship. For the Measurement or Fact Extension table, the Darwin Core elements included are: measurementID, eventID, measurementType, measurementValue, measurementUnit. This dataset is licensed under a Creative Commons Attribution (CC-BY 4.0) licence. The custodian of all the information collected is the Sierra Nevada Global-Change Observatory. The owner of the information collected by the Sierra Nevada Global-Change Observatory is the Department of Sustainability, Environment and Blue Economy (Regional Government of Andalusia), whereas the University of Granada is the owner of the data collected by its researchers.

## Technical Validation

- Digitalisation: data from the Sierra Nevada Global-Change Observatory monitoring methodology is digitalised in Linaria (https://linaria.obsnev.es/), the institutional data repository of the Sierra Nevada Global-Change Observatory, through data entry web forms, which includes several validation rules. Linaria is a normalised database focused on ecology and biodiversity related-data and it is developed in a PostgreSQL/PostGIS relational database management system (RDBMS) version 12.16^15^. Regarding the bibliographical data compilation, all data were extracted from the bibliographic database compiled by each of the experts throughout their research career, who have collected all the information that has been published on species present in the Sierra Nevada over the years.
- Data debugging: to check specific errors derived from digitalisation and correct them, several validations were performed. The following are highlighted:
  Taxonomic validation: scientific names were checked with the checklist of the arthropod fauna of the Sierra Nevada mountain range^16^ and subsequent taxonomic updates^17^. In addition, the scientific names of Plecoptera, Trichoptera and Coleoptera were reviewed by the expert authors.
  Locations validation: verbatim coordinates were converted from original formats (MGRS, UTM, sexagesimal degrees, etc.) to decimal degrees with the GBIF.ES coordinate converter tool (https://www.gbif.es/en/datos-biodiversidad/participa-en-gbif-es/herramientas-de-publicacion/). Sampling locations were checked with the study area and with official spatial data of rivers^18,19^. The *waterBody* Darwin Core element was completed with the names given by these official sources, in order to homogenise this information.
- Storage: all data is stored in Linaria.
- Standardisation: the standardisation to Darwin Core was done according to the practices recommended by the TDWG guidelines (https://dwc.tdwg.org/terms/). All data have been revised by experts before their publication in GBIF.

## Data Availability

No custom code has been used in this study.
